# Human UC-MSC-derived exosomes facilitate ovarian renovation in rats with chemotherapy-induced premature ovarian insufficiency

**DOI:** 10.3389/fendo.2023.1205901

**Published:** 2023-07-26

**Authors:** Xiaodi Pu, Leisheng Zhang, Peiyu Zhang, Yaqiong Xu, Jun Wang, Xiaomei Zhao, Zhihua Dai, Hua Zhou, Shuyun Zhao, Anran Fan

**Affiliations:** ^1^ Department of Obstetrics and Gynecology, Guizhou Medical University, Guiyang, China; ^2^ Stem Cell Bank of Guizhou Province, Guizhou Health-Biotech Biotechnology Co., Ltd., Guiyang, China; ^3^ Key Laboratory of Molecular Diagnostics and Precision Medicine for Surgical Oncology in Gansu Province, Gansu Provincial Hospital, Lanzhou, China; ^4^ National Health Commission (NHC) Key Laboratory of Diagnosis and Therapy of Gastrointestinal Tumor, Gansu Provincial Hospital, Lanzhou, China; ^5^ Key Laboratory of Radiation Technology and Biophysics, Hefei Institute of Physical Science, Chinese Academy of Sciences, Hefei, China; ^6^ Reproductive Medicine Center, Department of Obstetrics and Gynecology of the Affiliated Hospital of Guizhou Medical University, Guiyang, China; ^7^ Key Laboratory of Reproductive Medicine, Stem Cell and Tissue Engineering Research Center in Guizhou Province, Guizhou Medical University, Guiyang, China

**Keywords:** premature ovarian insufficiency (POI), HUCMSCs, exosomes, ovarian function, gene expression profile

## Abstract

Premature ovarian insufficiency (POI) induced by chemotherapy is an intractable disorder with a considerable incidence that commonly results in insufficient fertility and concomitant complications in female patients. Due to limitations in the current progress in POI diagnosis and treatment, there is an urgent need to develop novel remedies to improve ovarian function and protect fertility. The ameliorative effect of human umbilical cord mesenchymal stem cells (hUCMSCs) and exosomes derived from them in POI treatment could be a new hope for patients. Herein, we identified exosomes from hUCMSCs (hUCMSC-Exos). Then, systematic infusion of hUCMSC-Exos was accomplished via tail intravenous injection to investigate the feasibility of the treatment of rats with chemotherapy-induced POI by intraperitoneal injection of cyclophosphamide (CTX) and busulfan (BUS). Ovarian functions in the indicated group were evaluated, including oestrous cycle, serum sex hormone levels, follicle counts, ovarian pathological changes, proliferation and apoptosis of granulosa cells (GCs), and reproductive ability testing. Furthermore, the potential influence of hUCMSC-Exos on ovarian tissues was illuminated by conducting RNA-seq and multifaceted bioinformatics analyses. POI rats with hUCMSC-Exos transplantation exhibited a decrease in follicle-stimulating hormone (FSH) and apoptosis of GCs but an increase in oestradiol (E2), anti-Müllerian hormone (AMH), and the number of ovarian follicles and foetuses in the uterus. And the immunomodulation- and cellular vitality-associated gene sets in rats had also undergone moderate changes. Our data indicated the feasibility of hUCMSC-Exos in improving ovarian function and protecting fertility in chemotherapy-induced POI rats. HUCMSC-Exos can improve the local microenvironment of ovarian tissue in POI rats by participating in immune regulation, cellular viability, inflammation regulation, fibrosis and metabolism, and other related signal pathways.

## Introduction

Premature ovarian insufficiency (POI) refers to a clinical syndrome in which women younger than 40 years develop ovarian dysfunction, which is mainly characterized by abnormal menstruation (amenorrhea or oligomenorrhea for at least 4 months), increased gonadotropin levels (an elevated follicle-stimulating hormone (FSH) level > 25 IU/L on two occasions > 4 weeks apart), and oestrogen-deficiency symptom (1). POI is not a rare disease, with ESHRE reporting a global incidence of 1% in women under 40 years of age, but the actual incidence may be higher than reported. The World Health Organization’s International Agency for Research on Cancer (IARC) released new data on the global cancer burden in 2020. The latest estimates show that there were 19.29 million new cancer cases worldwide in 2020, 10.06 million in men, and 9.23 million in women (2), and the age of onset is getting younger. With the development of medical technology, the long-term survival rate of female cancer patients has been significantly improved through surgery, chemoradiotherapy, and other means. However, these treatments can lead to decreased ovarian function and even loss of fertility. With the long-term survival rate of female cancer survivors improving, they hope that anticancer treatments can better protect ovarian function and even fulfil unrealized reproductive needs. Chemotherapy is commonly used in the treatment of various malignant tumours, but it also has inevitable effects. Research has indicated that chemotherapy agents, such as CTX and BUS *in vivo*, trigger the growth of quiescent primordial follicles by upregulating *PI3K/Akt* signalling, resulting in the loss of the ovarian reserve, which occurs simultaneously with GC apoptosis (3). Thus, there is an urgent need to explore chemotherapy-induced POI and to develop safer and more effective strategies for POI management.

Mesenchymal stem/stromal cells (MSCs) have been acknowledged as advantageous sources for tissue engineering due to their unique characteristics, such as immunomodulation, haematopoietic-supporting effects, and multilineage differentiation potential (4, 5). MSCs of different origins revealed multifaceted variations in cellular vitality, immunomodulatory properties, and the resultant therapeutic efficacy in multiple diseases, as we and other investigators have previously reported (6, 7). Of them, human umbilical cord-derived MSCs (hUCMSCs) have been recognized to have the most robust cellular vitality and lower immunogenicity than their relative counterparts (6, 8, 9). MSCs function via a variety of approaches, such as trans- and direct-differentiation, secretion (paracrine, autocrine), dual immunomodulation, and even service as a constitutive microenvironment (10). For example, MSCs secrete a variety of vesicles, including exosomes and small extracellular vesicles (sEVs) (11–13). Exosomes are members of extracellular vesicles (60–150 nm in diameter) with bilayer lipid membranes and can be detected in a variety of biological fluids as well as in the supernatant of the cell culture medium (14). Exosomes mainly play a role by carrying paracrine factors secreted by hUCMSCs in exosomes, including non-coding RNA, cytokines, and growth factors, and then mediating intercellular communication and regulating target cell function (15). As reported by Fan et al. (16), exosomes have been shown to reduce chemotherapy-induced cardiotoxicity and liver damage by inhibiting apoptosis and repairing damaged tissues.

Therefore, in this study, we aimed to investigate the effects of hUCMSC-Exos transplantation on ovarian function and fertility in chemotherapy-induced POI rats from the point of view of biological manifestations and gene expression profiles. Collectively, our data also indicated that hUCMSC-Exos could remedy the ovarian function of POI rats and improve the local microenvironment of ovarian tissue in POI rats by participating in immune regulation, cellular viability, inflammation regulation, fibrosis, and metabolism.

## Materials and methods

### Identification of hUCMSC-Exos

hUCMSC-Exos (specification: 2 ml, purity ≥99%, it can be used for the study of the function mechanism of exosomes in cell and *in vivo* experiments; The exosomes derived from human umbilical cord mesenchymal stem cells we purchased are still in the experimental stage, and their components and the relevant content of specific proteomic analysis need to be kept secret and published together in the later project content.) were purchased from Guizhou Health-Biotech Co., Ltd. (China), isolated at a concentration of 1×10^10^ particles/ml and stored in a 4°C. The hUCMSC-Exos were experimentally identified as previously reported (17). First, we observed their morphologies by using transmission electron microscopy (TEM; Talos F200C; Thermo Scientific, US). Then, we used nanoparticle tracking analysis (NTA) to measure the exosomal size distribution and concentration. Finally, surface markers of hUCMSC-Exos, including CD9, CD63, and CD81 (Biolegend, US), were detected by western blot analysis.

### Animals

At 8 weeks of age, 40 female specific-pathogen-free (SPF)-grade Sprague-Dawley (SD) rats were purchased from Changsha Tianqin Biotechnology Co., Ltd. (China). After two weeks of adaptive feeding, 36 SD rats with stable oestrous cycles were selected for this study. All experimental procedures for animal handling were approved by the Animal Ethics Committee of the Affiliated Hospital of Guizhou Medical University (Approval number: 2101344).

### POI Rat model establishment and grouping

To establish the chemotherapy-induced POI model in rats (18, 19), 36 healthy female rats with stable oestrous cycles were randomly divided into the control, POI, and hUCMSC-Exos treatment groups (n = 12 in each group). The rats in the POI and hUCMSC-Exos treatment groups received a single intraperitoneal injection (IP) of cyclophosphamide (CTX, Shanghai Baxter Medical Supplies Trading Co., Ltd.) dissolved in 0.9% saline solution at a dose of 83.52 mg/kg combined with busulfan (BUS, C_6_H_14_O_6_S_2_, purity ≥ 98%, Beijing Solarbio Science & Technology Co., Ltd.) dissolved in dimethyl sulfoxide (DMSO) at a dose of 20.88 mg/kg (19). The rats in the control group were injected with an equivalent amount of normal saline by intraperitoneal injection. Beginning on the first day of chemotherapy, the rats in the three groups were weighed every week before being sacrificed.

### hUCMSC-Exo transplantation

At 24 h after chemotherapy as reported (19, 20), each rat from the hUCMSC-Exos group was injected with a volume of 100 μL purchased hUCMSC-Exos (1ml PBS containing 1×10^10^ particles hUCMSC-Exos) via the tail vein. The POI group was injected with an equivalent volume of normal saline via the tail vein, and the control group was also treated.

### Examination of oestrus cycles

After the CTX and BUS injections, vaginal smears of the rats in each group were obtained at 10:00 am daily for 4 weeks to observe the oestrous cycle (21). There are 4 consecutive stages in the normal oestrus cycle of rats: proestrus, oestrus, metestrus, and dioestrus. We identified the stages according to the presence or absence and the number of nucleated epithelial cells, keratinized epithelial cells, and leukocytes on the vaginal smears. A cotton swab moistened with normal saline was gently placed into the vagina, rotated 2-3 times, and then removed. The swab was evenly smeared on a glass slide, air-dried, and stained with 0.2% methylene blue dye solution. The smears of vaginal cells were observed under an optical microscope (Leica, Germany).

### ELISA assay

After 4 weeks of treatment, 24 rats (8 animals in each group) were anaesthetized with 10% chloral hydrate at a dose of 300 mg/kg by IP during a dioestrus period, and whole-blood samples were collected from the abdominal aorta. The blood samples were centrifuged at 3000 g for 10 minutes at 4°C after being left at room temperature for 2 hours, and the supernatants were collected and stored at -80°C until testing. Based on the manufacturer’s instructions, the serum levels of FSH, oestradiol (E2), and anti-Müllerian hormone (AMH) were measured with ELISA kits (Shanghai Xinfan Biological Technology Co., Ltd.).

### Ovarian follicle counts and histological analysis

After the animals were euthanized, the ovaries were collected, and some were fixed with 4% paraformaldehyde for 24 hours, dehydrated and embedded in paraffin for pathological analysis. The remaining ovaries were stored in RNAwait solution and then frozen at -80°C for further processing. The paraffin blocks were serially sectioned at a thickness of 5 μm, and the sections were stained with haematoxylin and eosin (H&E), after which the morphological structure of the ovaries was evaluated and the number of follicles were counted under an optical microscope. According to references, ovarian follicles are classified as primordial, primary, secondary, preovulatory, and atretic follicles (20, 22), and counted in every fifth section of the ovary (23).

### Immunohistochemical staining of PCNA

The proliferation of GCs in ovarian tissues of the Control, POI, and hUCMSC-Exos groups was detected using immunohistochemical staining of the cell proliferation marker PCNA. Antigen heat repair was performed with 0.01 mol/L sodium citrate buffer. Each sample was treated with 3% hydrogen peroxide and incubated at room temperature for 20 minutes. Then, the specific primary antibody was added, and the samples were incubated overnight at 4°C. The sections were incubated with HRP-conjugated goat anti-rabbit IgG polymer at 37 °C for 20 minutes. The sections were chromogenically developed with 3,3′-diaminobenzidine (DAB) after being washed in PBS. Finally, the slices were counterstained with haematoxylin for 2 minutes, gradually differentiated, rinsed, dehydrated, cleared, and sealed. The sections were finally observed with an optical microscope.

### Apoptosis assay

A one-step TUNEL cell apoptosis detection kit (Jiangsu Kaiji Biotechnology Co., Ltd.) was used to detect the disruption of nuclear DNA during apoptosis in ovarian tissues. According to the instructions provided by the manufacturer, the nuclei were counterstained with 4′, 6-diamidino-2-phenylindole dihydrochloride (DAPI), with the apoptotic cells in the ovary stained green (24). Finally, the images were captured by using fluorescence microscopy.

### Reproductive tests

To evaluate the fertility improvement of hUCMSC-Exos treatment, 4 weeks after hUCMSC-Exos transplantation, 4 randomly selected rats in each group were placed in a cage with sexually mature male rats at a 2:1 ratio, and the fertility levels of the three groups were recorded, including the number of foetuses in the uterus of rats in each group.

### RNA-seq and bioinformatics analyses

Four ovarian tissues from each of the indicated groups (Control, POI, and POI + hUCMSC-Exos) were used for total RNA extraction by using TRIzol reagent (ThermoFisher, USA) according to the manufacturer’s instructions as we reported before with several modifications (8, 25). RNA-seq analysis was conducted by BGI (Wuhan, China), and multifaceted bioinformatics analyses (e.g., Venn Map, HeatMap, Circos, KEGG, GOBP analyses) were accomplished by using online websites and databases as we recently described (25–27).

### Statistical analysis

At least 3 replicates were performed for each test in this study. All experimental data were processed with the professional statistical software SPSS 23.0. The results are presented as the mean ± standard deviation (SD) and were analysed by Student’s t test, one-way ANOVA, nonparametric Kruskal-Walli’s test, and Wilcoxon rank test for differences between experimental groups and within experimental groups. Statistical differences were indicated by *P* < 0.05. NS, not significant; *, *P*<0.05; **, *P*<0.01; ***, *P*<0.001.

## Results

### Identification of hUCMSC-Exos

**Figure 1:**
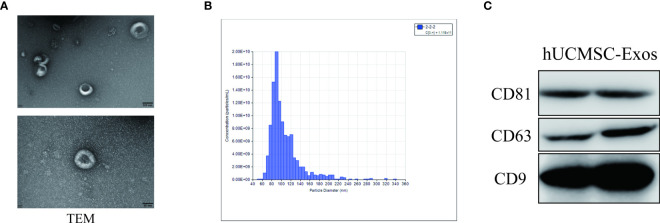
Identification of hUCMSC-Exos. **(A)** Representative ultrastructure of hUCMSC-derived exosomes (hUCMSC-Exos) under different magnifications by transmission electron microscopy (TEM). Scale bar=100 nm (upper panel), scale bar=50 nm (bottom panel). **(B)** Representative nanoparticle tracking analysis of the diameter of hUCMSC-Exos. **(C)** Western blotting analysis of exosome-related (CD9, CD63 and CD81) biomarkers in hUCMSC-Exos.

As recently reported (17, 26), a small portion of the purchased hUCMSC-Exos was removed and available for further identification of exosomes. As shown by nanoparticle tracking analysis, exosomes derived from hUCMSCs (denoted hUCMSC-Exos) revealed a discoid shape with particle diameters ranging from 60 to 150 nm (**Figures 1A, B**). Subsequently, by conducting a western blotting assay, we further confirmed the expression of typical exosome-specific biomarkers in hUCMSC-Exos, including CD9, CD63, and CD81 (**Figure 1C**). Collectively, the purchased hUCMSC-Exos were successfully identified.

### hUCMSC-Exos transplantation improved ovarian function in POI Rats

**Figure 2:**
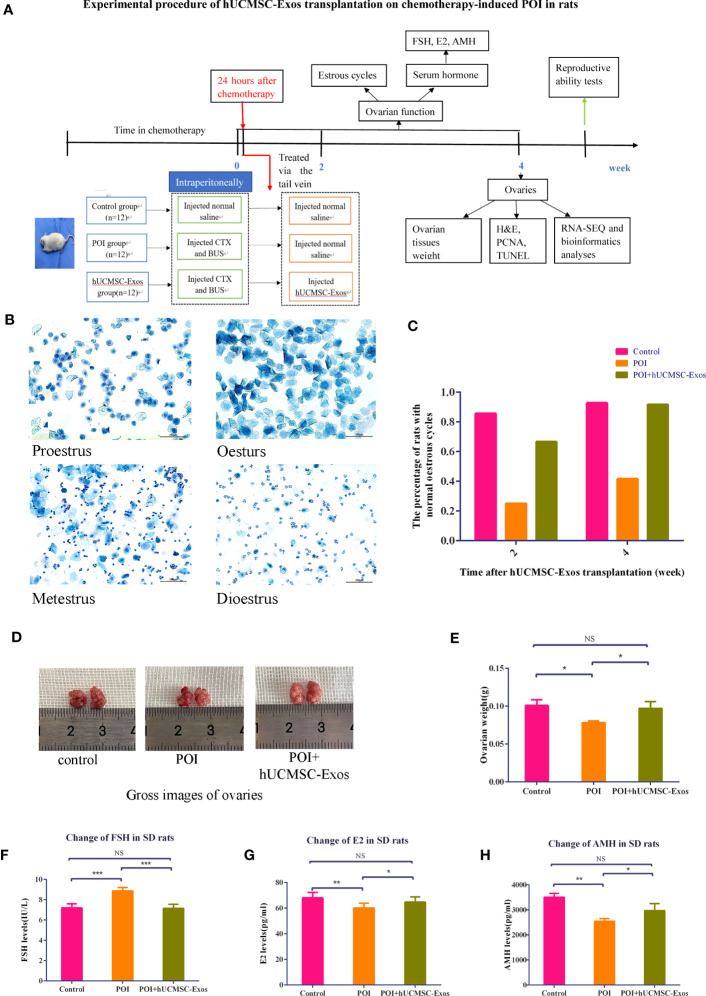
hUCMSC-Exos administration ameliorated ovarian function in chemotherapy-induced POI rats. **(A)** Illustration of chemotherapy-induced POI modelling and evaluation of hUCMSC-Exo-based treatment. **(B)** Representative images revealed variations in the oestrous cycle (including proestrus, oestrus, metestrus, and dioestrus) after staining with 0.2% methylene blue dye solution. Scale bar=100 μm. **(C)** The percentage of SD rats with normal oestrous cycles (2 or 4 weeks after hUCMSC-Exos administration) in the indicated groups. **(D)** Gross images of ovaries in the indicated groups (control, POI, and POI + hUCMSC-Exos). **(E)** Variations in ovarian weights of SD rats in the indicated groups. **(F–H)** Serum levels of FSH **(F)**, E2 **(G)**, and AMH **(H)** were measured at 4 weeks after hUCMSC-Exo administration. All data are shown as the mean ± SEM (n=4). *P<0.05; **P<0.01; ***P<0.001; NS, not significant.

To mimic the manifestations of POI, we took advantage of the chemotherapy-induced SD rat model as reported before. In more detail, POI rats in the experimental group were injected with hUCMSC-Exos through caudal vein (POI + hUCMSC-Exos), whereas the concomitant POI group was injected with an equal volume of normal saline (POI) (19) (**Figure 2A**). According to the methylene blue staining of the vaginal smears, we were able to observe the continuous progress of the oestrous cycle, including proestrus, oestrus, metestrus, and dioestrus (**Figure 2B**). Distinct from the rats in the control group (control), all POI rats showed a sharp decline in regular oestrous cycles before hUCMSC-Exo administration (Control vs. POI=92.8% vs. 25%). Instead, POI rats were observed to have irregular oestrous cycles after chemotherapy, including prolonged dioestrus periods, sustained or prolonged oestrous periods, and no periodicity. This indicates that we had successfully constructed an animal model of chemotherapy-induced POI. Notably, the percentage of POI rats with normal oestrous cycles increased to 91.6% two weeks after hUCMSC-Exo administration (**Figure 2C**). From the overview of the gross images, we observed minimal differences in ovarian size and morphology among the indicated groups (**Figure 2D**). However, POI rats revealed a moderate decrease in ovarian weight compared to the control group (*P*<0.05), which could be effectively rescued with hUCMSC-Exos treatment (**Figure 2E**). To further investigate the therapeutic effects of hUCMSC-Exos on POI rats, we detected the serum levels of hormones and found that the abnormally elevated levels of FSH in POI rats declined to a normal level in the POI + hUCMSC-Exos group (**Figure 2F**). Conversely, the levels of E2 and AMH in POI + hUCMSC-Exos rats were restored to those in the control group, which were higher than those in the POI group (*P*<0.05) (**Figures 2G, H**). Taken together, our data indicated the considerable efficacy of hUCMSC-Exos on the defect of ovarian function in POI rats.

### The pathological characteristics and increase in follicle counts were continuously ameliorated after hUCMSC-Exos infusion

**Figure 3:**
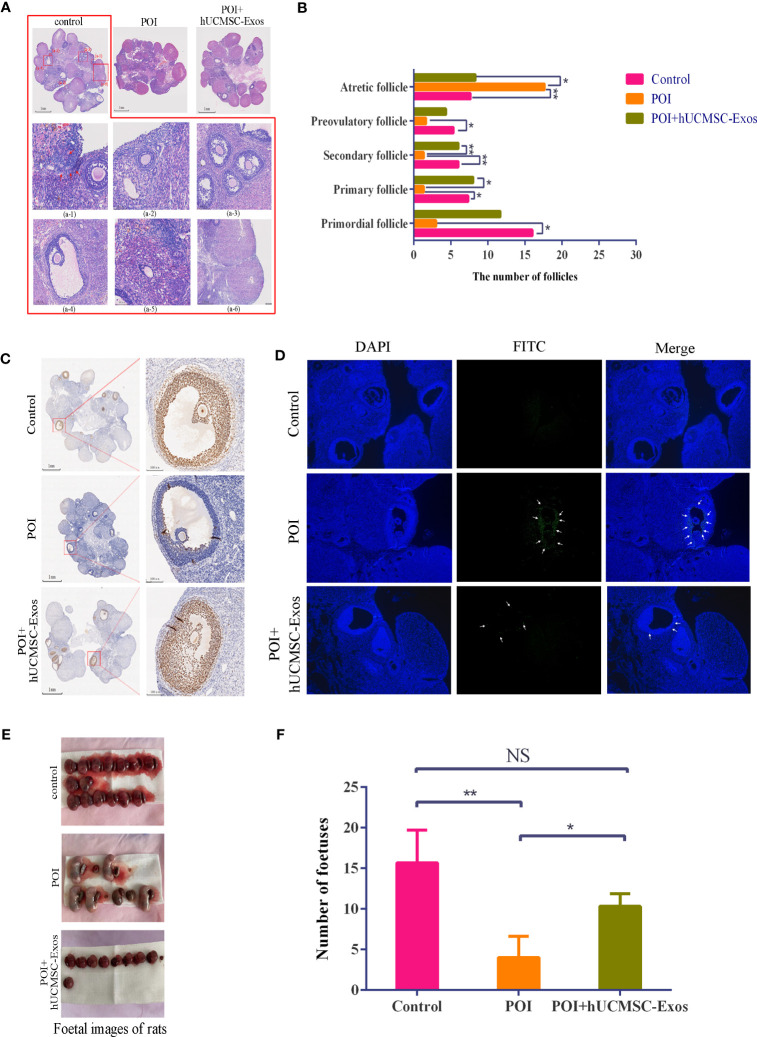
Variations in reproductive ability and histopathology after hUCMSC-Exos administration. **(A)** H&E staining revealed the histological variations in ovaries in the indicated groups. Scale bar=1 mm. Representative follicles from the control group are shown, including primordial follicles (a-1), primary follicles (a-2), secondary follicles (a-3), preovulatory follicles (a-4), atretic follicles (a-5), and corpus luteum (a-6). Scale bar=50 μm. **(B)** Statistical analysis of follicle numbers in the indicated groups. **(C)** PCNA analysis revealed the proliferation of granulosa cells in the ovaries in the indicated groups. Scale bar=1 mm (left panel), scale bar=100 μm (right panel). **(D)** Representative immunofluorescence images revealed cellular apoptosis in the ovaries in the indicated groups (x100). **(E, F)** The morphology **(E)** and number **(F)** of representative foetuses in the indicated groups. All data are shown as the mean ± SEM (n=4). *P<0.05; **P<0.01; NS, not significant.

To further evaluate the feasibility of hUCMSC-Exos injection for POI amelioration, we turned to histopathologic analysis and found that in the POI group, ovarian atrophy and tissue fibrosis were aggravated, and the number of follicles at various stages (primordial, primary, secondary, and preovulatory follicles) was significantly less than those in the control group. After hUCMSC-Exos injection, the number of follicles at various stages was significantly increased compared with that in the POI group (**Figures 3A, B**).

Furthermore, to verify the potential mechanism of hUCMSC-Exos for POI amelioration, we conducted PCNA immunohistochemistry and immunofluorescent staining to determine the proliferation and apoptosis of GCs. On the one hand, a declining number of proliferating cells was observed in the ovarian tissue of POI rats compared with the control group, which was effectively alleviated with hUCMSC-Exos injection (**Figure 3C**). On the other hand, a higher proportion of apoptotic cells detected with FITC-based immunofluorescence was observed in POI rats than in the other groups (**Figure 3D**). Taken together, these data indicated the therapeutic effect of hUCMSC-Exos on ovarian insufficiency by modulating cellular proliferation and apoptosis in POI rats.

To fundamentally assess the ameliorative effect of hUCMSC-Exos on POI, we turned to fertility analysis based on the statistical calculation of fetuses *in utero*. Notably, the number of fetuses in the POI group was sharply decreased compared to that in the control group, whereas there was a vast increase with hUCMSC-Exos treatment (POI vs. hUCMSC-Exos =23.4% vs. 65.9%) (*P*<0.05) (**Figures 3E, F**). Compared with the POI group, the number of fetuses in the POI + hUCMSC-Exos group increased significantly, and their reproductive capacity returned to near the control levels, so there was no significant difference in statistics between the control group and the POI +hUCMSC-Exos group (**Figure 3F**). Overall, hUCMSC-Exo injection was adequate to alleviate pathological characteristics and improve the reproductive performance of POI rats.

### Gene expression profiling of ovarian tissue revealed variations in POI rats

**Figure 4:**
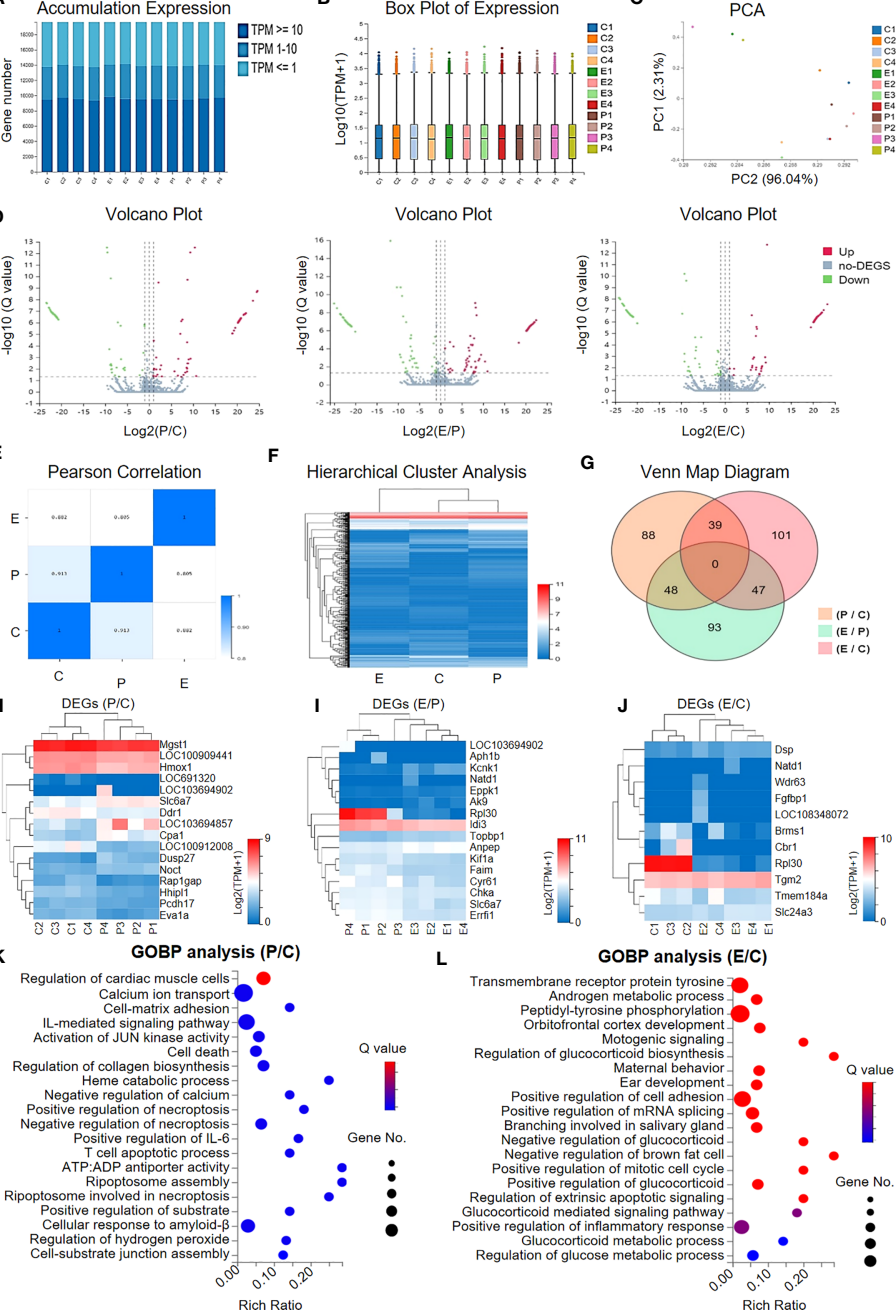
Gene expression profiling of the granular cells in the indicated groups. **(A, B)** The accumulation **(A)** and box plot **(B)** of gene expression in the granular cells in the indicated groups (C, P, E) based on the TPM values. **(C)** Principal component analysis (PCA) of the individuals in the three groups based on TPM. **(D)** Volcano plots of the gene expression profiling between the indicated groups (P vs. C, E vs. P, E vs. C) including the upregulated genes (denoted as Up), the downregulated genes (denoted as Down), and those non-differentially expressed genes (denoted as no-DEGs). **(E)** Pearson correlation of the groups. **(F)** Hierarchical cluster analysis showed the gene expression profiling in the groups. **(G)** Venn Map diagram showing the DEGs among the indicated groups. **(H–J)** Heatmap diagrams showing the DEGs between the indicated groups, including P vs. C **(H)**, E vs. P **(I)**, and E vs. C **(J)**. **(K, L)** Gene Ontology Biological Process (GOBP) analysis of the DEGs between the P group and the C group **(K)** and between the E group and the C group **(L)**.

Having clarified the therapeutic effect of hUCMSC-Exos on the recovery of ovarian function, we next became curious about the potential impacts on ovarian cells in ovarian tissue at the molecular level. For this purpose, we used RNA-seq and bioinformatics analyses to further dissect the potential similarities and differences in gene expression profiling. As shown by the accumulation chart and box plot, we observed conservation and similarities in gene expression patterns based on transcripts per million (TPM) values (**Figures 4A, B**). According to the principal component analysis (PCA), we did not observe distinct grouping among the indicated individuals in the control group (denoted as C1, C2, C3, and C4), the POI group (denoted as P1, P2, P3, and P4), and the POI + hUCMSC-Exos group (denoted as E1, E2, E3, and E4) (**Figure 4C**). Simultaneously, by conducting volcano plot analysis based on the Q value, the distribution of the upregulated (Up) and downregulated (Down) differentially expressed genes (DEGs) and the non-DEGs (no-DEGs) was intuitively shown (**Figure 4D**).

The genetic relationship among the indicated groups (C, P, and E) was also available according to Pearson correlation and hierarchical cluster analysis (**Figures 4E, F**). As shown by the Venn Map diagram, a total of 416 DEGs with more than a 2-fold change in gene expression between the indicated groups were observed (**Figure 4G**). For instance, a cluster of representative genes involved in immunoregulation (e.g., *Cpa1*, *Brms1*), fibrosis (e.g., *Hmox1*, *Eva1a*, *Eppk1*, *Errfl1*, *Fgfbp1*), cellular vitality (cell growth, differentiation, programmed cell death) and metabolism (e.g., *Ddr1*, *Pcdh17*, *Noct*, *Rpl30*, *Cbr1*) were enriched among the indicated groups (**Figures 4H–J**). With the aid of Gene Ontology Biological Process (GOBP) analysis, we verified that the DEGs between P and C were mainly involved in cellular vitality (e.g., cell death, necroptosis, apoptosis) and immunomodulation (e.g., IL-mediated signalling pathway), whereas those between E and C were principally related to metabolism (e.g., androgen metabolic process, glucocorticoid and glucose metabolic process) and cellular vitality (e.g., mitotic cell cycle, extrinsic apoptotic signalling) (**Figures 4K, L**). Taken together, our data indicated that hUCMSC-Exos facilitated the recovery of ovarian function by ameliorating inflammatory responses and promoting cellular vitality without significantly impacting gene expression profiling in the ovarian tissue of POI rats.

### hUCMSC-Exo infusion benefited ovary renovation and influenced on VSEs in POI rats

**Figure 5:**
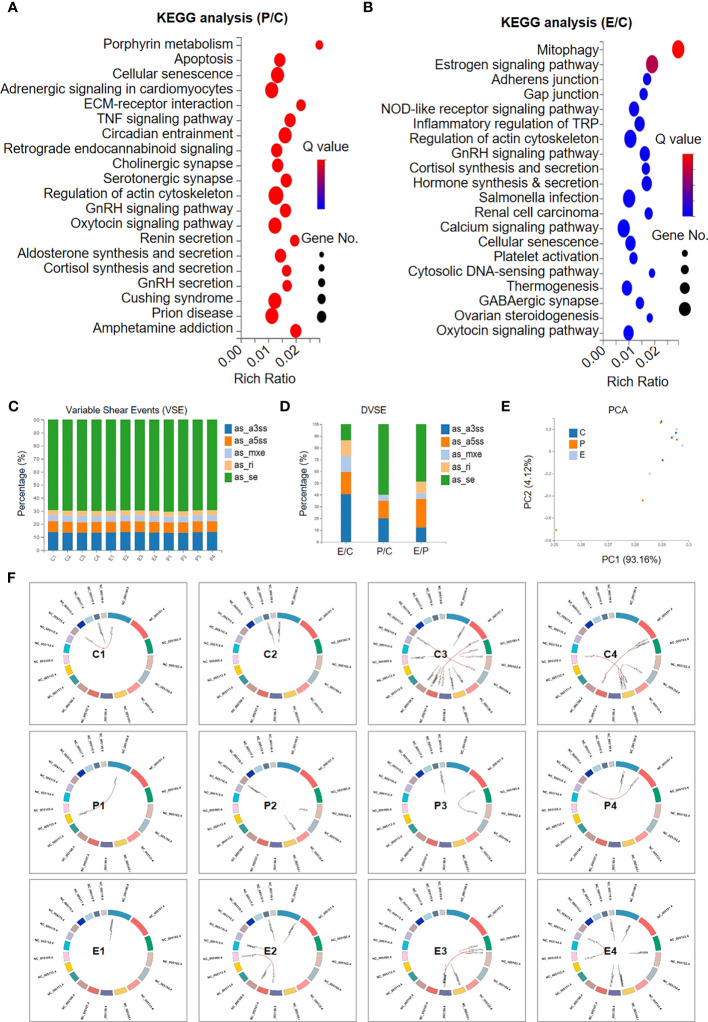
Signalling pathway analysis and variable shear events between the indicated groups. **(A, B)** KEGG analysis revealed the involvement of the DEGs in multiple signalling pathways between the P group and the C group **(A)** and between the E group and the C group **(B)**. **(C, D)** The percentages of the subsets of variable shear events (VSEs) **(C)** and the differential VSEs (DVSEs) **(D)** in the indicated groups. **(E)** The PCA diagram shows the individuals in the aforementioned groups based on VSE. **(F)** Circos diagrams reveal the variations in the regional distribution of VSE loci and gene fusion events in the indicated groups.

To further illuminate the potential influence of hUCMSC-Exo infusion on POI rats, we used KEGG analysis of DEGs between the indicated rats. Regarding the P and C groups, the DEGs were mainly involved in cellular vitality-, immunoregulation-, and interaction-associated signals, such as cellular senescence and apoptosis, the TNF signalling pathway, the GnRH signalling pathway, ECM-receptor interactions, and the actin cytoskeleton, which collectively suggested the impairment of ovarian tissue in POI rats (**Figure 5A**). Interestingly, we found that a series of gene subsets were involved in ovarian renovation-associated bioprocesses, including synthesis and sectioning, ovarian steroidogenesis, inflammatory regulation, and cellular senescence, which collectively suggested the therapeutic effect of hUCMSC-Exos on POI rats (**Figure 5B**).

Having verified the DEGs and the underlying biological significance, we next focused on the potential impact of hUCMSC-Exos on variable shear events (VSEs) in POI rats, as we reported previously (28). The bar chart indicated the similarity in VSEs among the individuals, including as_se, as_ri, as_mxe, as_a3ss, and as_a5ss (**Figure 5C**). Among them, a few genes showed significant differences in VSEs (differential VSEs, DVSEs) among groups. We can see that the proportion of as_se variable cutting events increased significantly between P and C, and decreased significantly between E and C with hUCMSC-Exos treatment, while the trend of as_mxe, as_a3ss, and as_a5ss variable cutting events was just the opposite (**Figure 5D**). On PCA, we found that there was no significant difference in VSEs distribution among the three groups (**Figure 5E**). Furthermore, by conducting Circos analysis, the distributions of the genes with genetic variations were intuitively exhibited, and no consistent variations were observed in the individuals of the C, P, and E groups (**Figure 5F**). However, this cannot completely prove that hUCMSC-Exos treatment has no significant effect on the genetic variation of ovarian tissue in POI rats. Taken together, our data suggest that hUCMSC-Exos works by improving the local microenvironment of POI rat ovarian tissue through signaling pathways related to immune regulation, cellular viability, inflammation regulation, fibrosis, and metabolism.

## Discussion

An intractable disorder causing the cessation of ovarian function before 40 years of age, POI has been acknowledged to reduce life expectancy and quality of life (29). For decades, we and other investigators in the field have been devoted to illuminating the pathogenesis of POI and developing novel therapeutic remedies, yet the efficient amelioration of POI is still challenging. Herein, we took advantage of the chemotherapy-induced POI rat model to verify the feasibility of hUCMSC-Exos injection for improving ovarian function and protecting fertility in POI rats and further investigate the potential mechanism of exosomes promoting ovarian function recovery in POI rats.

POI has been recognized as a frequent and problematic complication of surgery with an incidence ranging from 10% to 30% and is considered to be associated with several aetiologies, including autoimmune, genetic, and iatrogenic categories (30). For example, autoimmunity has been reported to be responsible for 4% to 30% of POI cases, and X chromosome aberrations (e.g., duplications, deletions, and X-autosome rearrangements) also show correlations with the POI phenotype (e.g., *Xq13-Xq21* to *Xq23-Xq27*) *(* 31). Notably, chemotherapy, a common treatment for various malignancies, has been believed to induce ovarian failure and hamper fertility in young females, including approximately 30% and 50% of women before 35 years of age and between 35 and 40 years of age, respectively (32). Currently, hormone replacement therapy (HRT), such as oestrogen, progestogens, and androgens, has been recommended for women with POI, particularly for those with genitourinary and vasomotor symptoms (33), but the reproductive function of their ovaries has not been fundamentally improved. However, no current therapy has been proven to be effective for POI patients who receive high-dose radiotherapy and chemotherapy (20).

Current studies have shown that 42% of female cancer patients treated with alkylating agents will further develop into POI (32). CTX and BUS, both of which are alkylating agents, are highly ovarian toxic and can alter cellular DNA to destroy cells, which may cause inhibition of primordial follicle proliferation and may directly contribute to follicle depletion and oocyte loss, leading to ovarian dysfunction and even premature ovarian failure (POF) (34, 35). CTX combined with BUS can better simulate the performance of POI in the POI model induced by SD rats, and it has also been found that cancer survivors treated with alkylating agents have the highest risk of POI (36). Therefore, we successfully established a chemotherapy-induced POI rat model by using CTX with BUS.

MSCs are heterogeneous cell populations with multipotent lineage differentiation potential and immunoregulatory properties that have been considered to play a critical role in the haematopoietic microenvironment and regenerative medicine. State-of-the-art literature has suggested the mitigative effect of MSC-based cytotherapy in a variety of refractory and recurrent diseases, such as acute myeloid leukaemia (AML), Crohn’s disease (37), aplastic anaemia (8, 25), osteoarthritis (5), rheumatoid arthritis (38) and COVID-19-related acute respiratory distress syndrome (ARDS) (39). Currently, pioneers in the field have also investigated the feasibility of MSCs for the management of reproductive diseases, including POF and POI (40). For example Ling et al. (20) verified the efficacy of human amnion-derived MSC (hAD-MSC) transplantation in improving ovarian function in rats with POI, which is mainly mediated by paracrine effect of MSC-derived exosomes. The hUCMSC-Exos used in this study were identified as being consistent with the structures of exosomes mentioned in some literature (41). In animal experiments on the treatment of many diseases, hUCMSC-Exos not only shows similar biological function to MSCs but also has more stable biological activity *in vivo*, a lower content of exosomes membrane binding protein, a lower possibility of immune rejection, no proliferation ability, no risk of tumorigenesis, the source is simple and uncontroversial, it is easy to preserve, and the dose and concentration are easy to control (42, 43). Therefore, it has a broad application prospect in the field of acellular therapy.

In this study, we found that hUCMSC-Exos could improve ovarian function and reproductive capacity in POI rats by promoting GC proliferation and reducing GC apoptosis in follicles. To further confirm the role of hUCMSC-Exos in POI rats, we constructed a POI rat model using CTX and BUS and then injected hUCMSC-Exos into POI rats through the caudal vein. We observed that the oestrous cycle of POI rats gradually recovered, serum sex hormone levels (FSH, E2, and AMH) returned to near normal levels, the number of follicles increased, the number of atretic follicles decreased, the proliferation of GCs in the follicles decreased, and GC apoptosis decreased. It is well known that GCs and oocytes are important components of follicles, and FSH is a necessary hormone for follicle development and is responsible for follicle growth. E2 collaborates with FSH to promote follicle development, and the E2 level in turn provides negative feedback on pituitary FSH secretion on the hypothalamic-pituitary-ovarian axis. AMH can be secreted by GCs in primary and secondary follicles, and the number of primordial follicles directly affects the serum AMH level and inhibits the initiation of primordial follicle growth. More and more studies have shown that hUCMSC-Exos also play an important role in regulating hormone secretion in the ovary (44, 45). Therefore, these three hormones have been considered as the best indicators for the early detection of POI (20, 46). Excessive apoptosis of GCs is the key mechanism of follicular atresia, which can lead to follicular dysfunction and ovarian physiological changes. This suggests that promoting GC proliferation can alleviate impaired ovarian morphology and function (47). In addition, exploring the therapeutic effect of hUCMSC-Exos in GCs could help identify the potential mechanism of exosome-related therapy in POI. Herein, our data provide direct evidence that hUCMSC-Exos ameliorate the pathological manifestations and insufficiency of fertility in POI rats.

Improving the reproductive function of the ovaries is another important goal of POI therapy. To verify the efficacy of hUCMSC-Exos therapy, female and male rats in each group were caged in a 2:1 ratio after hUCMSC-Exos transplantation, and their fertility was observed. The results showed that the reproductive function of our POI rats was significantly improved with hUCMSC-Exos transplantation. In short, after hUCMSC-Exos transplantation, the number of foetuses in the utero of POI rats increased significantly. Therefore, we can reasonably speculate that after hUCMSC-Exos injection, the proliferation of GCs in the primordial and primary follicles of POI rats can be promoted, the oestrous cycle and serum sex hormone levels can be restored, the apoptosis of GCs can be inhibited, and the development of follicles can be promoted, thus improving the fertility of POI rats (48).

Meanwhile, there are few reports on the mechanism of hUCMSC-Exos in the treatment of POI disease through gene expression profiles and enriched signal pathways, which is of great importance for dissecting the aetiology and the concomitant development of novel remedies (49). With the aid of RNA-seq and multifaceted bioinformatics analyses, we found that these DEGs between P and C or between E and C are mainly involved in immune regulation, cellular viability, fibrosis, metabolism, and other related activities. In the P and C groups, DEGs were increased in cell senescence and apoptosis, the TNF signaling pathway, the IL-mediated signaling pathway, the GnRH signaling pathway, ECM receptor interaction, and the actin cytoskeleton, which collectively suggested the damage to ovarian tissue in POI rats. The aging and apoptosis of GCs are the main reasons for the decline of ovarian function, and the status of GCs in the ovary is very important for the development of follicles. Reducing the apoptosis of GCs in the ovary and promoting the repair of the original follicle reserve pool are the main mechanisms to restore ovarian function (50). The increased expression of TNF and IL inflammatory factors indicated that the occurrence of POI was closely related to the inflammatory response. hUCMSC-Exos transplantation can improve ovarian function in POI rats by inhibiting anti-inflammatory effects through the TNF signaling pathway and the IL-mediated signaling pathway. The GnRH signaling pathway affects the growth, development, and ovulation of ovarian follicles through hormone secretion. In groups E and C, we identified a series of gene subsets involved in biological processes related to ovarian gene repair, including synthesis and sectioning, ovarian steroid production, hormone synthesis and secretion, inflammation regulation, and cell senescence, which collectively suggested that hUCMSC-Exos showed therapeutic effects on POI rats. The synthesis of ovarian steroid hormones is involved in the regulation of follicles and the maintenance of ovarian reproductive function, which is closely related to ovarian development and aging (51). By comparing the DVSEs between the control group and the POI group, we realized that as_se was significantly increased in POI rats, which might be one of the causes of POI. Our study also indicated that the therapeutic effect of hUCMSC-Exos on POI rats was mainly due to the amelioration in local microenvironment in ovarian tissue, including cellular vitality, inflammation, immune regulation, fibrosis and metabolism. To sum up, our data provide a new strategy for the treatment of infertility in female patients with POI, which also provides a theoretical basis for the clinical diagnosis and treatment of POI disease with hUCMSC-Exos as cell-free therapy in future.

This study laid an experimental foundation for further preclinical trials of hUCMSC-Exos in the treatment of POI, but there are still some shortcomings. CTX combined with BUS is a one-time modeling method. Because of the large dose during intraperitoneal injection and the high requirements for laboratory personnel, improper operation will increase the mortality of experimental animals. It is necessary to increase the sample size and expand the groups to further explore the optimal therapeutic dose, administration time, and administration route of hUCMSC-Exos to improve POI rats, as well as the effect on the birth of cubs after modeling. Secondly, we did not label the implanted hUCMSC-Exos because of concerns about whether it would return to the damaged ovarian tissue as scheduled and whether its biological activity *in vivo* would be altered. Therefore, in our follow-up study, we need to include more animals to observe the therapeutic effect of hUCMSC-Exos at different times or in model POI through *in vivo* and *in vitro* experiments so as to fully understand its mechanism.

## Conclusions

Overall, our data demonstrated that hUCMSC-Exo transplantation was adequate to alleviate ovarian injury, facilitate ovarian function restoration, and protect fertility in chemotherapy-induced POI rats. The results of RNA-seq sequencing and bioinformatics analysis show that hUCMSC-Exos plays a role in improving the local microenvironment of ovarian tissue in POI rats through immune regulation, cellular viability, inflammation regulation, fibrosis, and metabolism. Our findings suggest that the new strategy based on hUCMSC-Exos may be applied to the treatment of POI disease in the future.

## Author contributions

XP, LZ: experiment design and performance, collection and assembly of data, and manuscript writing; PZ, YX, JW, XZ and ZD: literature search; HZ, SZ and AF: conception and design, revision, final approval of manuscript. All authors have read and approved the final manuscript.

## References

[1] European Society for Human R Embryology Guideline Group on POI Webber L Davies M Anderson R Bartlett J. ESHRE Guideline: management of women with premature ovarian insufficiency. Hum Reprod (2016) 31(5):926–37. doi: 10.1093/humrep/dew027 27008889

[2] Sung H Ferlay J Siegel RL Laversanne M Soerjomataram I Jemal A. Global Cancer Statistics 2020: GLOBOCAN estimates of incidence and mortality worldwide for 36 cancers in 185 countries. CA Cancer J Clin (2021) 71(3):209–49. doi: 10.3322/caac.21660 33538338

[3] Kalich-Philosoph L Roness H Carmely A Fishel-Bartal M Ligumsky H Paglin S. Cyclophosphamide triggers follicle activation and "burnout"; AS101 prevents follicle loss and preserves fertility. Sci Transl Med (2013) 5(185):185ra162. doi: 10.1126/scitranslmed.3005402 23677591

[4] Volarevic V Gazdic M Simovic Markovic B Jovicic N Djonov V Arsenijevic N. Mesenchymal stem cell-derived factors: Immuno-modulatory effects and therapeutic potential. Biofactors (2017) 43(5):633–44. doi: 10.1002/biof.1374 28718997

[5] Zhang L Wei Y Chi Y Liu D Yang S Han Z. Two-step generation of mesenchymal stem/stromal cells from human pluripotent stem cells with reinforced efficacy upon osteoarthritis rabbits by HA hydrogel. Cell Biosci (2021) 11(1):6. doi: 10.1186/s13578-020-00516-x 33407870 PMC7787598

[6] Zhao Q Zhang L Wei Y Yu H Zou L Huo J. Systematic comparison of hUC-MSCs at various passages reveals the variations of signatures and therapeutic effect on acute graft-versus-host disease. Stem Cell Res Ther (2019) 10(1):354. doi: 10.1186/s13287-019-1478-4 31779707 PMC6883552

[7] Lin BL Chen JF Qiu WH Wang KW Xie DY Chen XY. Allogeneic bone marrow-derived mesenchymal stromal cells for hepatitis B virus-related acute-on-chronic liver failure: A randomized controlled trial. Hepatology (2017) 66(1):209–19. doi: 10.1002/hep.29189 28370357

[8] Wei Y Zhang L Chi Y Ren X Gao Y Song B. High-efficient generation of VCAM-1(+) mesenchymal stem cells with multidimensional superiorities in signatures and efficacy on aplastic anaemia mice. Cell Prolif (2020) 53(8):e12862. doi: 10.1111/cpr.12862 32597552 PMC7445411

[9] Ding DC Chang YH Shyu WC Lin SZ. Human umbilical cord mesenchymal stem cells: a new era for stem cell therapy. Cell Transplant (2015) 24(3):339–47. doi: 10.3727/096368915X686841 25622293

[10] Zhang L Wang H Liu C Wu Q Su P Wu D. MSX2 initiates and accelerates mesenchymal stem/stromal cell specification of hPSCs by regulating TWIST1 and PRAME. Stem Cell Rep (2018) 11(2):497–513. doi: 10.1016/j.stemcr.2018.06.019 PMC6092836 30033084

[11] Zhang H Wang L Li C Yu Y Yi Y Wang J. Exosome-induced regulation in inflammatory bowel disease. Front Immunol (2019) 10:1464. doi: 10.3389/fimmu.2019.01464 31316512 PMC6611439

[12] Lai JJ Chau ZL Chen SY Hill JJ Korpany KV Liang NW. Exosome processing and characterization approaches for research and technology development. Adv Sci (Weinh) (2022) 9(15):e2103222. doi: 10.1002/advs.202103222 35332686 PMC9130923

[13] Wei Y Hou H Zhang L Zhao N Li C Huo J. JNKi- and DAC-programmed mesenchymal stem/stromal cells from hESCs facilitate hematopoiesis and alleviate hind limb ischemia. Stem Cell Res Ther (2019) 10(1):186. doi: 10.1186/s13287-019-1302-1 31234947 PMC6591900

[14] Alenquer M Amorim MJ. Exosome biogenesis, regulation, and function in viral infection. Viruses (2015) 7(9):5066–83. doi: 10.3390/v7092862 PMC4584306 26393640

[15] Xu S Liu C Ji HL. Concise review: therapeutic potential of the mesenchymal stem cell derived secretome and extracellular vesicles for radiation-induced lung injury: progress and hypotheses. Stem Cells Transl Med (2019) 8(4):344–54. doi: 10.1002/sctm.18-0038 PMC6431606 30618085

[16] Fan M Li H Shen D Wang Z Liu H Zhu D. Decoy exosomes offer protection against chemotherapy-Induced toxicity. Adv Sci (Weinh) (2022) 9(32):e2203505. doi: 10.1002/advs.202203505 36058003 PMC9661835

[17] Thery C Witwer KW Aikawa E Alcaraz MJ Anderson JD Andriantsitohaina R. Minimal information for studies of extracellular vesicles 2018 (MISEV2018): a position statement of the International Society for Extracellular Vesicles and update of the MISEV2014 guidelines. J Extracell Vesicles (2018) 7(1):1535750. doi: 10.1080/20013078.2018.1535750 30637094 PMC6322352

[18] Luo Q Yin N Zhang L Yuan W Zhao W Luan X. Role of SDF-1/CXCR4 and cytokines in the development of ovary injury in chemotherapy drug induced premature ovarian failure mice. Life Sci (2017) 179:103–9. doi: 10.1016/j.lfs.2017.05.001 28478265

[19] Feng X Ling L Zhang W Liu X Wang Y Luo Y. Effects of human amnion-derived mesenchymal stem cell (hAD-MSC) transplantation *in situ* on primary ovarian insufficiency in SD rats. Reprod Sci (2020) 27(7):1502–12. doi: 10.1007/s43032-020-00147-0 31953773

[20] Ling L Feng X Wei T Wang Y Wang Y Wang Z. Human amnion-derived mesenchymal stem cell (hAD-MSC) transplantation improves ovarian function in rats with premature ovarian insufficiency (POI) at least partly through a paracrine mechanism. Stem Cell Res Ther (2019) 10(1):46. doi: 10.1186/s13287-019-1136-x 30683144 PMC6347748

[21] Eldridge JC Wetzel LT Tyrey L. Estrous cycle patterns of Sprague-Dawley rats during acute and chronic atrazine administration. Reprod Toxicol (1999) 13(6):491–9. doi: 10.1016/S0890-6238(99)00056-8 10613397

[22] Myers M Britt KL Wreford NG Ebling FJ Kerr JB. Methods for quantifying follicular numbers within the mouse ovary. Reproduction (2004) 127(5):569–80. doi: 10.1530/rep.1.00095 15129012

[23] Wang Z Wang Y Yang T Li J Yang X. Study of the reparative effects of menstrual-derived stem cells on premature ovarian failure in mice. Stem Cell Res Ther (2017) 8(1):11. doi: 10.1186/s13287-016-0458-1 28114977 PMC5259841

[24] Yin N Zhao W Luo Q Yuan W Luan X Zhang H. Restoring ovarian function with human placenta-derived mesenchymal stem cells in autoimmune-induced premature ovarian failure mice mediated by Treg cells and associated cytokines. Reprod Sci (2018) 25(7):1073–82. doi: 10.1177/1933719117732156 28954601

[25] Huo J Zhang L Ren X Li C Li X Dong P. Multifaceted characterization of the signatures and efficacy of mesenchymal stem/stromal cells in acquired aplastic anemia. Stem Cell Res Ther (2020) 11(1):59. doi: 10.1186/s13287-020-1577-2 32054519 PMC7020384

[26] Sun Y Wang TE Hu Q Zhang W Zeng Y Lai X. Systematic comparation of the biological and transcriptomic landscapes of human amniotic mesenchymal stem cells under serum-containing and serum-free conditions. Stem Cell Res Ther (2022) 13(1):490. doi: 10.1186/s13287-022-03179-2 36195964 PMC9530421

[27] Zhang L Liu M Song B Miao W Zhan R Yang S. Decoding the multidimensional signatures of resident and expanded natural killer cells generated from perinatal blood. Am J Cancer Res (2022) 12(5):2132–45. doi: 10.1186/s13287-022-03008-6 PMC9185604 35693070

[28] Zhang L Chi Y Wei Y Zhang W Wang F Zhang L. Bone marrow-derived mesenchymal stem/stromal cells in patients with acute myeloid leukemia reveal transcriptome alterations and deficiency in cellular vitality. Stem Cell Res Ther (2021) 12(1):365. doi: 10.1186/s13287-021-02444-0 34174939 PMC8233618

[29] Tsiligiannis S Panay N Stevenson JC. Premature ovarian insufficiency and long-term health consequences. Curr Vasc Pharmacol (2019) 17(6):604–9. doi: 10.2174/1570161117666190122101611 30819073

[30] Laven JS. Primary ovarian insufficiency. Semin Reprod Med (2016) 34(4):230–4. doi: 10.1055/s-0036-1585402 27513024

[31] Barros F Carvalho F Barros A Doria S. Premature ovarian insufficiency: clinical orientations for genetic testing and genetic counseling. Porto BioMed J (2020) 5(3):e62. doi: 10.1097/j.pbj.0000000000000062 PMC7722400 33299945

[32] Meirow D Nugent D. The effects of radiotherapy and chemotherapy on female reproduction. Hum Reprod Update (2001) 7(6):535–43. doi: 10.1093/humupd/7.6.535 11727861

[33] Webber L Anderson RA Davies M Janse F Vermeulen N. HRT for women with premature ovarian insufficiency: a comprehensive review. Hum Reprod Open (2017) 2017(2):hox007. doi: 10.1093/hropen/hox007 30895225 PMC6276684

[34] Detti L Martin DC Williams RW Schlabritz-Loutsevich N Williams LJ Uhlmann RA. Somatic and reproductive outcomes in mice treated with cyclophosphamide in pre-pubertal age. Syst Biol Reprod Med (2013) 59(3):140–5. doi: 10.3109/19396368.2012.751463 23278118

[35] Ezoe K Murata N Yabuuchi A Okuno T Kobayashi T Kato O. Long-term adverse effects of cyclophosphamide on follicular growth and angiogenesis in mouse ovaries. Reprod Biol (2014) 14(3):238–42. doi: 10.1016/j.repbio.2014.04.007 25152523

[36] Overbeek A van den Berg MH van Leeuwen FE Kaspers GJ Lambalk CB van Dulmen-den Broeder E. Chemotherapy-related late adverse effects on ovarian function in female survivors of childhood and young adult cancer: A systematic review. Cancer Treat Rev (2017) 53:10–24. doi: 10.1016/j.ctrv.2016.11.006 28056411

[37] Hou H Zhang L Duan L Liu Y Han Z Li Z. Spatio-temporal metabolokinetics and efficacy of human placenta-derived mesenchymal stem/stromal cells on mice with refractory Crohn's-like enterocutaneous fistula. Stem Cell Rev Rep (2020) 16(6):1292–304. doi: 10.1007/s12015-020-10053-2 33011925

[38] Lopez-Santalla M Fernandez-Perez R Garin MI. Mesenchymal stem/stromal cells for rheumatoid arthritis treatment: an update on clinical applications. Cells (2020) 9(8):1852. doi: 10.3390/cells9081852 32784608 PMC7465092

[39] Zhang LS Yu Y Yu H Han ZC. Therapeutic prospects of mesenchymal stem/stromal cells in COVID-19 associated pulmonary diseases: From bench to bedside. World J Stem Cells (2021) 13(8):1058–71. doi: 10.4252/wjsc.v13.i8.1058 PMC8422925 34567425

[40] Shareghi-Oskoue O Aghebati-Maleki L Yousefi M. Transplantation of human umbilical cord mesenchymal stem cells to treat premature ovarian failure. Stem Cell Res Ther (2021) 12(1):454. doi: 10.1186/s13287-021-02529-w 34380572 PMC8359553

[41] Purushothaman A. Exosomes from Cell Culture-Conditioned Medium: Isolation by Ultracentrifugation and Characterization. Methods Mol Biol (2019) 1952:233–244. doi: 10.1007/978-1-4939-9133-4_19 30825179

[42] Pluchino S Smith JA. Explicating exosomes: reclassifying the rising stars of intercellular communication. Cell (2019) 177(2):225–7. doi: 10.1016/j.cell.2019.03.020 30951665

[43] Johnson TV Bull ND Martin KR. Identification of barriers to retinal engraftment of transplanted stem cells. Invest Ophthalmol Vis Sci (2010) 51(2):960–70. doi: 10.1167/iovs.09-3884 PMC2868445 19850833

[44] Dai A Sun H Fang T Zhang Q Wu S Jiang Y. MicroRNA-133b stimulates ovarian estradiol synthesis by targeting Foxl2. FEBS Lett (2013) 587(15):2474–82. doi: 10.1016/j.febslet.2013.06.023 23810756

[45] Cai JH Sun YT Bao S. HucMSCs-exosomes containing miR-21 promoted estrogen production in ovarian granulosa cells via LATS1-mediated phosphorylation of LOXL2 and YAP. Gen Comp Endocrinol (2022) 321-322:114015. doi: 10.1016/j.ygcen.2022.114015 35271888

[46] Silva MSB Giacobini P. New insights into anti-Müllerian hormone role in the hypothalamic-pituitary-gonadal axis and neuroendocrine development. Cell Mol Life Sci (2021) 78(1):1–16. doi: 10.1007/s00018-020-03576-x PMC7867527 32564094

[47] Li Z Zhang M Zheng J Tian Y Zhang H Tan Y. Human umbilical cord mesenchymal stem cell-derived exosomes improve ovarian function and proliferation of premature ovarian insufficiency by regulating the hippo signaling pathway. Front Endocrinol (Lausanne) (2021) 12:711902. doi: 10.3389/fendo.2021.711902 34456868 PMC8397419

[48] Zhang S Huang B Su P Chang Q Li P Song A. Concentrated exosomes from menstrual blood-derived stromal cells improves ovarian activity in a rat model of premature ovarian insufficiency. Stem Cell Res Ther (2021) 12(1):178. doi: 10.1186/s13287-021-02255-3 33712079 PMC7953711

[49] Ishizuka B. Current understanding of the etiology, symptomatology, and treatment options in premature ovarian insufficiency (POI). Front Endocrinol (Lausanne) (2021) 12:626924. doi: 10.3389/fendo.2021.626924 33716979 PMC7949002

[50] Liu M Qiu Y Xue Z Wu R Li J Niu X. Small extracellular vesicles derived from embryonic stem cells restore ovarian function of premature ovarian failure through PI3K/AKT signaling pathway. Stem Cell Res Ther (2020) 11(1):3. doi: 10.1186/s13287-019-1508-2 31900201 PMC6942273

[51] da Silveira JC Winger QA Bouma GJ Carnevale EM. Effects of age on follicular fluid exosomal microRNAs and granulosa cell transforming growth factor-beta signalling during follicle development in the mare. Reprod Fertil Dev (2015) 27(6):897–905. doi: 10.1071/RD14452 25945781

